# RAP-011 Rescues the Disease Phenotype in a Cellular Model of Congenital Dyserythropoietic Anemia Type II by Inhibiting the SMAD2-3 Pathway

**DOI:** 10.3390/ijms21155577

**Published:** 2020-08-04

**Authors:** Gianluca De Rosa, Immacolata Andolfo, Roberta Marra, Francesco Manna, Barbara Eleni Rosato, Achille Iolascon, Roberta Russo

**Affiliations:** 1 Department of Molecular Medicine and Medical Biotechnologies, University of Naples Federico II, 80131 Naples, Italy; gianlucadr88@gmail.com (G.D.R.); robertamarra.r@gmail.com (R.M.); rosato.barbara@gmail.com (B.E.R.); achille.iolascon@unina.it (A.I.); 2 Ceinge Biotecnologie Avanzate, 80145 Naples, Italy; manna@ceinge.unina.it

**Keywords:** congenital dyserythropoietic anemia type II, activin receptor II ligand trap, in vitro drug treatment

## Abstract

Congenital dyserythropoietic anemia type II (CDA II) is a hypo-productive anemia defined by ineffective erythropoiesis through maturation arrest of erythroid precursors. CDA II is an autosomal recessive disorder due to loss-of-function mutations in *SEC23B*. Currently, management of patients with CDA II is based on transfusions, splenectomy, or hematopoietic stem-cell transplantation. Several studies have highlighted benefits of ACE-011 (sotatercept) treatment of ineffective erythropoiesis, which acts as a ligand trap against growth differentiation factor (GDF)11. Herein, we show that GDF11 levels are increased in CDA II, which suggests sotatercept as a targeted therapy for treatment of these patients. Treatment of stable clones of *SEC23B*-silenced erythroleukemia K562 cells with the iron-containing porphyrin hemin plus GDF11 increased expression of pSMAD2 and reduced nuclear localization of the transcription factor GATA1, with subsequent reduced gene expression of erythroid differentiation markers. We demonstrate that treatment of these *SEC23B*-silenced K562 cells with RAP-011, a “murinized” ortholog of sotatercept, rescues the disease phenotype by restoring gene expression of erythroid markers through inhibition of the phosphorylated SMAD2 pathway. Our data also demonstrate the effect of RAP-011 treatment in reducing the expression of erythroferrone in vitro, thus suggesting a possible beneficial role of the use of sotatercept in the management of iron overload in patients with CDA II.

## 1. Introduction

Congenital dyserythropoietic anemias (CDAs) are a group of rare hereditary disorders that are defined by maturation arrest of the erythroid lineage, the main consequence of which is ineffective erythropoiesis, with the consequent reduced production of erythrocytes [1]. CDA type II (CDA II) is the most common form of CDAs, and it is caused by biallelic loss-of-function mutations in the *SEC23B* gene. *SEC23B* encodes a protein of the COPII complex that is involved in intracellular vesicle trafficking from the endoplasmic reticulum to the Golgi compartment [2,3]. CDA II is characterized by normocytic anemia of variable degree and relative reticulocytopenia, which are often accompanied by jaundice and splenomegaly, due to the hemolytic component. Bone-marrow examination of patients with CDA II has highlighted erythroid hyperplasia and the presence of bi-nucleated and multi-nucleated erythroblasts, due to maturation arrest [1,4]. The most harmful complication for patients with CDA II is iron overload [5]. Indeed, ineffective erythropoiesis results in strong downregulation of the hepatic hormone hepcidin, and this condition can lead to increased iron absorption and systemic iron overload, which is mediated by the erythroid hormone erythroferrone (ERFE) [6].

In the last years, members of the transforming growth factor-beta (TGF-β) superfamily, which includes activins (A and B), growth differentiation factors (GDFs) and bone morphogenetic proteins (BMPs), have been studied as potential regulators of erythropoiesis. In particular, GDF11 (also known as BMP11) has been investigated as a possible negative regulator of erythropoiesis. Indeed, through its binding to activin receptors (ActR) IIA and IIB, GDF11 can inhibit terminal erythroid maturation [7]. Nevertheless, a recent study excluded that GDF11 is the only effector of TGF- inhibition of late erythropoiesis in mice, but it may contribute together with other related cytokines to ineffective erythropoiesis [8].

To date, treatments for patients with CDA II have only included supportive therapies, such as transfusion, splenectomy and hematopoietic stem-cell transplantation in transfusion-dependent patients [1,4,9,10]. Of note, two inhibitors of TGF- pathway, ACE-011 (sotatercept) and ACE-536 (luspatercept), are ligand traps for ActRIIA and ActRIIB, respectively, and their use has been associated with improved hematologic parameters in healthy post-menopausal women [11]. Studies with the “murinized” orthologs of sotatercept and luspatercept, known as RAP-011 and RAP-536, respectively, in a murine model of β-thalassemia resulted in increased differentiation of erythroid cells, improved anemia and reduced iron overload in the treated mice [11,12]. Sotatercept and its murinized counterpart RAP-011 are chimeric proteins where the extracellular domain of the ActRIIA receptor has been fused to the Fc portion of the human (or mouse, respectively) IgG1 antibody [13]. RAP-011 treatment has already been evaluated in a β-thalassemia murine model, as well as in a zebrafish model of Diamond–Blackfan anemia [7,13].

Here, we investigated the effects of RAP-011 treatment in an in vitro CDA II model of the erythroleukemia K562 cell line stably silenced for *SEC23B* expression.

## 2. Results

### 2.1. Ex Vivo and In Vitro Quantitative Evaluation of GDF11

**Figure 1:**
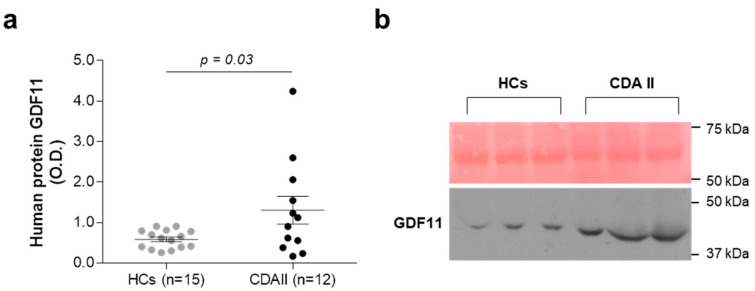
Ex vivo analysis of GDF11 expression. (**a**) Densitometry quantification of GDF11 expression in plasma samples from healthy controls (HCs; *n* = 15) and patients with CDA II (*n* = 12) on red ponceau-stained membrane. O.D., optical density. Data are means ± standard error. *p*-value by Mann–Whitney tests. (**b**) Representative Western blot for GDF11 expression in plasma samples from 3 healthy controls and 3 patients with CDA II.

On the basis of data that correlate increased GDF11 levels to β-thalassemia, we investigated the expression of this cytokine in healthy controls and patients with CDA II. In total, 15 healthy controls and 12 patients with *SEC23B*-related CDA II provided peripheral blood for evaluation of protein expression of GDF11. The ex vivo analysis at the protein level showed 2.2-fold increased expression of secreted GDF11 in plasma samples from the patients with CDA II compared to the healthy controls (Figure 1a,b).

K562 sh-*SEC23B*-74 and K562 sh-*SEC23B*-70 cells that were both stably silenced for *SEC23B* were induced to erythroid differentiation by hemin treatment (Figure S1a,b). Five days after this treatment, there was no significant increase of GDF11 levels in the K562 sh-*SEC23B*-74 cells compared to the K562 sh-CTR cells, and there was a significant marked increase in GDF11 in the K562 sh-*SEC23B*-70 cells (Figure S1c,d). Of note, the two silenced clones showed different behaviors in terms of expression of *SEC23A*, the paralog of *SEC23B*. Indeed, at steady-state, there was significant downregulation of *SEC23A* in the K562 sh-*SEC23B*-70 cells, which suggested a less-specific effect of the silencing in the K562 sh-*SEC23B*-70 cells (Figure S2). For this reason, we chose K562 sh-*SEC23B*-74 cells for the subsequent analyses.

### 2.2. SMAD2 Protein Phosphorylation is Inhibited by RAP-011

**Figure 2:**
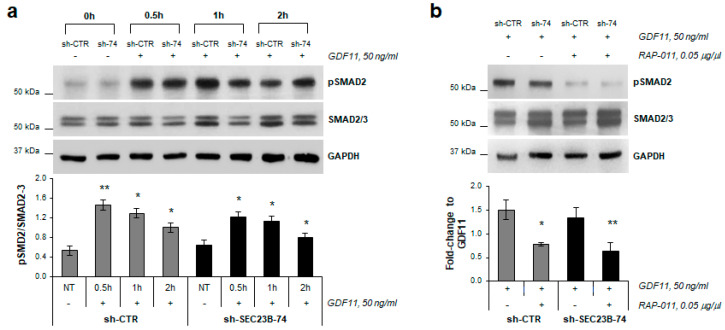
Effects of RAP-011 on SMAD2 pathway. (**a**) Representative Western blots and statistical analysis based on densitometric quantification of three replicates of phosphorylated SMAD2 (pSMAD2) for K562 sh-CTR and K562 sh-*SEC23B*-74 (sh-74) cells treated with GDF11 for 0 (NT), 0.5, 1 and 2 h (normalized to total SMAD2/3). (**b**) Representative Western blots and statistical analysis based on densitometric quantification of three replicates of phosphorylated SMAD2 (pSMAD2) for K562 sh-CTR and K562 sh-*SEC23B*-74 (sh-74) cells treated with GDF11 and GDF11 + RAP-011 (fold-change of pSMAD2 for GDF11 + RAP-011 compared to GDF11). Data are means ± standard deviations. *, *p* < 0.05; **, *p* < 0.01 (Student *t*-tests).

To reproduce the microenvironment of the CDA II marrow, human recombinant GDF11 was added to the cell medium at different times (0.5, 1, and 2 h) after two days of differentiation with hemin. There was an increase in the phosphorylation of SMAD2 in the K562 GDF11-treated cells compared to the non-treated cells (Figure 2a). Comparison of the GDF11-treated cells and the GDF11 + RAP-011-treated cells showed that the RAP-011 treatment led to significant reduction in the levels of the phosphorylated SMAD2 induced by GDF11 (Figure 2b).

### 2.3. RAP-011 Treatment Induces Nuclear Translocation of the Transcription Factor GATA1

**Figure 3:**
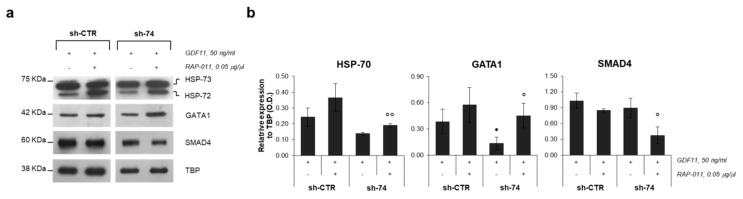
GATA1 nuclear localization after RAP-011 treatment. (**a**) Representative Western blot of three replicates of GATA1, HSP70 (HSP-73, constitutive isoform; HSP-72, inducible isoform) and SMAD4 localization in the nuclear compartment in K562 sh-CTR and K562 sh-*SEC23B*-74 (sh-74) cells treated with GDF11 and GDF11 + RAP-011 (normalized to TBP). (**b**) Densitometry quantification of Western blots of GATA1, HSP70, and SMAD4 localization in the nuclear compartment in K562 sh-CTR and K562 sh-*SEC23B*-74 (sh-74) cells treated with GDF11 and GDF11 + RAP-011 (normalized to TBP). Data are means ± standard deviations. •, *p* ≤ 0.05 (sh-CTR + GDF11 vs. sh-74 + GDF11); °, *p* ≤ 0.05, °°, *p* < 0.01 (sh-74 + GDF11 vs. sh-74 + GDF11/RAP-011) (Student *t*-tests).

To investigate the mechanism through which RAP-011 improved this erythroid differentiation, subcellular fractionation of the K562 sh-CTR and sh-*SEC23B*-74 cells was performed. There was increased expression of GATA1 in the nuclear compartment after the GDF11 + RAP-011 treatment for both the K562 sh-CTR and K562 sh-*SEC23B*-74 cells, but in particular for this *SEC23B*-silenced K562 cell clone, which showed a significant 3.4-fold increase. Similarly, there was increased expression of the molecular chaperone HSP70 in the nuclear fractions of the GDF11 + RAP-011-treated cells. Conversely, immunoblotting highlighted significantly decreased expression of SMAD4, the nuclear mediator of phospho-SMAD2 signaling, in both the K562 sh-CTR and K562 sh-*SEC23B*-74 cells treated with GDF11 + RAP-011, compared to those treated with GDF11 alone (Figure 3a,b).

### 2.4. GATA1 Nuclear Translocation Promoted by RAP-011 Restores Gene Expression of Erythroid Markers

**Figure 4:**
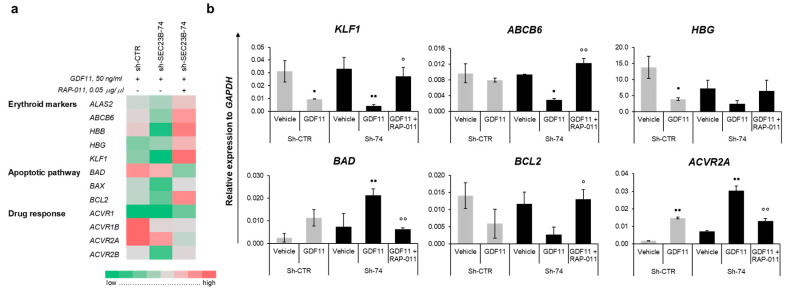
Gene expression profiling in RAP-011-treated cells. (**a**) Heat map for expression profiling for each of the cell clones (as indicated) treated with GDF11 or GDF11 + RAP-011. Fold-changes of sh-CTR and sh-*SEC23B*-74 cells treated with GDF11 were calculated on sh-CTR and sh-*SEC23B*-74 cells treated with vehicle. Fold-changes of sh-*SEC23B*-74 cells treated with GDF11 + RAP-011 were calculated on sh-*SEC23B*-74 cells treated with GDF11. Gene expression: green, low; grey, medium; red, high. A gray-scale version of the heat map is shown in Figure S3. (**b**) Relative expression of *KLF1*, *ABCB6*, *HBG*, *BAD*, *BCL2* and *AVR2A* genes in K562 sh-CTR and K562 sh-*SEC23B*-74 (sh-74) cells treated with GDF11 and GDF11 + RAP-011 (normalized to *GAPDH* gene). Data are means ± standard deviations of three experiments. *p*-value by ANOVA test, internal post-hoc correction by Tukey’s multiple comparison tests. •, *p* < 0.05; ••, *p* < 0.01 (vehicle vs. GDF11 treated cells); °, *p* < 0.05; °°, *p* < 0.01 (GDF11 treated cells vs. GDF11 + RAP-011 treated cells).

The responses to the GDF11 and RAP-011 treatments were then analyzed in terms of the expression profiles of the different genes involved in erythroid differentiation, apoptosis and the GDF11-response pathways. The erythroid differentiation markers *KLF1*, *ABCB6*, *ALAS2,* and *HBG* were markedly increased in the K562 sh-*SEC23B*-74 cells treated with GDF11 + RAP-011, compared to those treated with GDF11 alone. Moreover, there was rescued expression of *BCL2* in the K562 sh-*SEC23B*-74 cells treated with GDF11 + RAP-011, compared to those treated with GDF11 alone. Conversely, the K562 sh-*SEC23B*-74 cells showed downregulation of *BAX* and *BAD* after GDF11 + RAP-011 treatment. Finally, expression of the activin receptor genes was also downregulated in the K562 sh-*SEC23B*-74 cells treated with GDF11 + RAP-011 (Figure 4a,b).

### 2.5. RAP-011 Treatment Impairs Erythroferrone Expression

**Figure 5:**
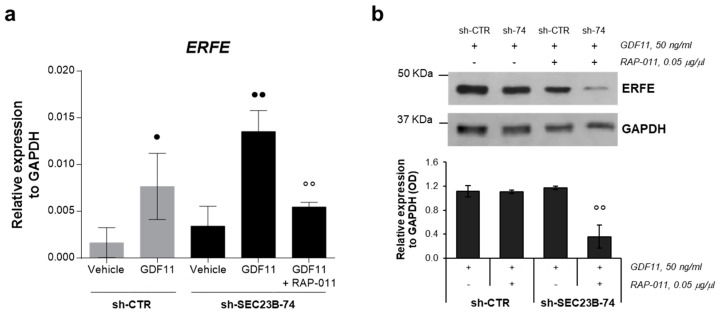
ERFE gene and protein expression in RAP-011-treated cells. (**a**) Quantification of *ERFE* in K562 sh-CTR and K562 sh-*SEC23B*-74 (sh-74) cells treated with GDF11 and GDF11 + RAP-011. Data are means ± standard deviations of three experiments. *p*-value by ANOVA test, internal post-hoc correction by Tukey’s multiple comparison tests. •, *p* < 0.05; ••, *p* < 0.01 (vehicle vs. GDF11 treated cells); °°, *p* < 0.01 (GDF11 treated cells vs. GDF11 + RAP-011 treated cells). (**b**) Representative Western blot of three replicates and densitometry quantification of ERFE expression in K562 sh-CTR and sh-*SEC23B*-74 (sh-74) cells treated with GDF11 and GDF11 + RAP-011 (normalized to GAPDH). Data are means ± standard deviations. *p* value by Student t-test; •, *p* < 0.05 (sh-CTR + GDF11 vs. sh-74 + GDF11); °°, *p* < 0.01 (sh-74 + GDF11 vs. sh-74 + GDF11 + RAP-011).

To investigate the effects of RAP-011 on the expression of ERFE, it was analyzed at both gene and protein levels. There was strong upregulation of *ERFE* expression in the K562 sh-*SEC23B*-74 cells treated with GDF11. Conversely, addition of RAP-011 also resulted in significantly marked reduction of *ERFE* expression in these K562 sh-*SEC23B*-74 cells (Figure 5a). Accordingly, marked downregulation of the ERFE protein levels was seen in these K562 sh-*SEC23B*-74 cells following the GDF11 + RAP-011 treatment (Figure 5b).

## 3. Discussion

Blood transfusion therapy or treatments with erythropoiesis-stimulating agents such as recombinant erythropoietin are the present front-line therapies for anemia associated with ineffective erythropoiesis. However, both treatments have side effects, and they are not always effective. Therefore, there is the clinical need for novel compounds with different mechanisms of action to those that are already available.

Recently, two inhibitors of TGF- pathway, sotatercept and luspatercept, have been evaluated for the treatment of hereditary anemias, such as β-thalassemia and Diamond–Blackfan anemia [7,13]. Sotatercept antagonizes GDF11, which is a negative regulator of erythropoiesis, as well as several other members of the TGF-β superfamily that signal through ActRIIA [14,15,16]. Of note, aberrant expression of GDF11 was demonstrated in a β-thalassemia murine model [7]. Although the early reports have identified GDF11 as the primary target of ligand traps [7,12], a recent study demonstrated that Hbb^th3/+^ and Hbb^+/+^ mice deleted for *Gdf11* are still able to respond to the ligand trap RAP-536, thus suggesting that Gdf11 is not the only effector of TGF- inhibition of late erythropoiesis in mice [8]. Nevertheless, this is in agreement with the observation that the ligand traps are able to bind also other members of the TGF- family, including GDF8 and activin B [11]. Additionally, we cannot exclude a different genetic or epigenetic regulation in human and mouse genes, as described for similar erythroid regulators as GDF15 [17]. It is conceivable to hypothesize that GDF11 is one of the players involved in the regulation of terminal erythroid differentiation.

Recently, a phase II clinical trial with sotatercept was carried out with patients with β-thalassemia, which produced encouraging results [18]. As β-thalassemia and CDA II show similar pathophysiologies, we first investigated the GDF11 levels in patients with CDA II. This analysis highlighted overexpression of GDF11 in the patients with CDA II, compared to healthy controls, which identified this biomarker as a possible therapeutic target for CDA II. Given the lack of a reliable mouse models for CDA II [19], we used the previously developed in vitro model of K562 cells stably silenced for *SEC23B* [5] to investigate the efficacy of RAP-011 treatment. Unlike ex vivo analysis, we did not observe any marked increase in GDF11 levels in these *SEC23B*-silenced K562 cells when they were induced to erythroid differentiation by hemin, compared to the control K562 cells. This difference might be due to the absence of systemic production of GDF11 in the K562 cell line. Given the increased expression of GDF11 in ineffective erythropoiesis, we treated these K562 cells with this cytokine to simulate the pathological context and analyze the effects of the RAP-011 treatment in our cellular model.

It has already been demonstrated that GDF11 acts through its binding to ActRIIA or ActRIIB, with the consequent phosphorylation of the intracellular mediator SMAD2 and activation of the SMAD2/ SMAD3/ SMAD4 complex. In our in vitro cell system, GDF11 treatment of both control K562 cells and *SEC23B*-silenced K562 cells resulted in increased phosphorylation of SMAD2 at different times, as expected, whereas the combined treatments with GDF11 and RAP-011 produced inhibition of the GDF11-ActR binding that led to reduced phosphorylation of SMAD2.

To understand how inhibition of the SMAD2 pathway improves erythroid survival, we investigated the role of the transcription factor GATA1 during this RAP-011 treatment. Arlet and colleagues demonstrated that, in β-thalassemia, the cytosol to nucleus translocation of GATA1 mediated by HSP70 is counteracted by the increased need of HSP70 for folding of denatured proteins. This process induces caspase-3-mediated cleavage of GATA1, and the consequent impairment of erythroid gene expression, end-stage maturation arrest and apoptosis [20].

Accordingly, we observed decreased expression of GATA1 and HSP70 in the nuclear compartment of cells treated with GDF11 alone versus with GDF11 plus RAP-011. Moreover, we observed decreased SMAD4 in the nuclear compartment of these RAP-011-treated cells, which confirmed that the RAP-011 treatment inhibited the GDF11-activated pathway [21].

We observed the downregulation of various erythroid markers in *SEC23B*-silenced K562 cells treated with GDF11. This was in agreement with Dussiot and colleagues, who demonstrated that treatment with recombinant GDF11 of bone marrow- and spleen-derived erythroblasts blocked terminal erythroblast maturation in thalassemic cells [7]. Thus, to evaluate the effects of RAP-011 on the transcription factor activity of GATA1, we analyzed gene expression of the several GATA1-induced erythroid markers: *HBG*, *KLF1*, *ALAS2* and *ABCB6*. In agreement with the enhanced nuclear translocation of GATA1, we observed increased expression of these erythroid marker genes in the *SEC23B*-silenced K562 cells treated with RAP-011.

Furthermore, to determine whether the addition of RAP-011 treatment restored caspase-3-mediated cleavage of GATA1, we also evaluated gene expression of proapoptotic and antiapoptotic genes, namely *BAX*, *BAD* and *BCL2*. Accordingly, after the RAP-011 treatment, we observed reduced expression of both *BAX* and *BAD* and overexpression of *BCL2*. These effects were directly correlated to the RAP-011 treatment. Indeed, the expression levels of the ActRs (types I, IB, IIA, and IIB) were reduced in the RAP-011-treated cells.

Iron overload due to reduced expression of hepatic hormone hepcidin is one of the main hallmarks of CDA II. As key erythroid regulator of pathological suppression of hepcidin expression, ERFE is overexpressed in CDA II patients and plays an important role in abnormal erythropoiesis [5]. Indeed, we recently described a low-frequency *ERFE* variant that is recurrent in patients with CDA II with a severe phenotype and that is associated with increased ERFE expression [22]. Thus, we investigated the effect of RAP-011 on ERFE expression as a biomarker of ineffective erythropoiesis. We observed increased ERFE expression in the *SEC23B*-silenced K562 cells after the GDF11 treatment. In the cells treated with GDF11 plus RAP-011, this GDF11-induced increased ERFE expression was blocked by addition of RAP-011 treatment and further reduced. This suggests that treatment with RAP-011 might have effects on the iron overload by reducing the levels of ERFE. These results are also in agreement with the phase II clinical trial based on sotatercept for patients with β-thalassemia [18]. Indeed, both transfusion-dependent and non-transfusion-dependent patients showed good responses in terms of increased hemoglobin levels, with reduced red blood cell transfusions for the transfusion-dependent patients, and finally also reduced systemic iron overload [18].

Here, we demonstrated that treatment with RAP-011, a murinized analog of sotatercept, can rescue the disease phenotype in GDF11-treated *SEC23B*-silenced K562 cells by restoring the expression of erythroid marker genes through inhibition of the phosphorylated SMAD2 pathway. Indeed, inhibition of GDF11-signaling pathway appears to translate into more intense transcriptional activity of these erythroid markers, which might allow undifferentiated erythroblasts to overcome the maturation arrest. These data also demonstrate the beneficial role of RAP-011 treatment for reduction of expression of ERFE, which again supports the use of sotatercept in the management of iron overload for patients with CDA II.

## 4. Materials and Methods

### 4.1. Patients

Twelve patients with CDA II and 15 age- and gender-matched healthy controls were enrolled in the study. CDA II diagnosis was based on clinical findings and biochemical and molecular analyses (Table S1), as previously reported [4,5,22,23]. The Naples University Ethical Committee (protocol number: 252/18, September 2018) approved the collection of the patient data from the Medical Genetics Ambulatory in Naples (DAIMedLab, “Federico II” University, Naples, Italy). Samples from the patients were obtained after signed informed consent, and according to the Declaration of Helsinki.

### 4.2. Production of Lentiviral Particles and Infection of the K562 Cell Line

Knock-down of SEC23B expression was obtained through infection of lentiviral particles that targeted human *SEC23B*, in human myeloid leukemia K562 cells, as previously described [5]. Briefly, we used pGIPZ Lentiviral shRNAmir targeting human *SEC23B* from Open Biosystems (Horizon Discovery, Waterbeach, UK). We used two different sh-RNAs for *SEC23B* (V3LHS_357970, sh-70; V3LHS_357974, sh-74) (Table S2). A non-silencing pGIPZ Lentiviral shRNAmir was used as control (RHS4346, sh-CTR). HEK-293T were transfected by 10 µg of sh-RNA plasmid DNA, 30 µL of Trans-Lentiviral Packaging Mix (Open Biosystems Horizon Discovery, Waterbeach, UK) and 25 µL of TransFectin (BioRad, Milan, Italy) in 10-mm plate. The supernatants (10 mL for points) were harvested after 24 h, centrifuged at low speed to remove cell debris and filtered through a 0.45-μm filter. After 48 h of incubation, the transduced cells were examined microscopically for the presence of TurboGFP expression (90–95%). The lentiviral particles of sh-CTR, sh-70 and sh-74 (50 MOI) were used to infect K562 cell line. After 48 h of infection, the cells were maintained in puromycin (0.5 mg/mL) for 2 weeks and then analyzed for GFP+ expression and sorted by cell sorting flow cytometry assay. The sorted GFP+ cells were then assayed for SEC23B expression to verify the stability of the produced clones.

### 4.3. Cell Culture and RAP-011 Treatment

The wild-type (control) K562 cells and *SEC23B*-silenced K562 stable clones were maintained in RPMI medium (Sigma Aldrich, Milan, Italy) supplemented with 10% fetal bovine serum (Sigma Aldrich, Milan, Italy) and grown in a humidified 5% CO_2_ incubator at 37 °C. Erythroid differentiation of the K562 cells was performed as previously described [24]. Briefly, 50 µM of the iron-containing porphyrin hemin (Sigma Aldrich, Milan, Italy) was added to the culture medium containing the wild-type (control) K562 cells (Sh-CTR) and the *SEC23B*-silenced K562 cell clones (Sh-*SEC23B*-70 and Sh-*SEC23B*-74 cells) (at 4 × 10^5^ cells/mL). Cell samples were collected at specific times: before hemin addition, on day 0 and on days 2 and 5 after hemin addition. Differentiation was assessed by FACS detection of the transferrin receptor 1 (CD71) [24]. Recombinant human GDF11 protein (1958-GD; R&D Systems, Minneapolis, MN, USA) was used at 50 ng/mL, with RAP-011 at 0.05 g/L (Celgene Corporation, Summit, NJ, USA).

### 4.4. Gene Expression Analysis

#### 4.4.1. RNA Isolation and Reverse Transcription.

Total RNA was extracted from peripheral blood cells and K562 cells using Trizol reagent (Life Technologies, Waltham, MA, USA). Synthesis of cDNA from total RNA (1 μg) was performed using cDNA synthesis kits (Life Technologies, Waltham, MA, USA Roche).

#### 4.4.2. Quantitative Real-Time PCR Analysis.

Quantitative real-time PCR (qRT-PCR) analysis was carried out using Power SYBR Green PCR Master Mix (Life Technologies, Waltham, MA, USA) to evaluate gene expression of the *GDF11*, *SEC23B*, *GATA1*, *KLF1*, *ABCB6*, *ALAS2*, *HBG*, *BCL*-2, *BAX*, *BAD*, *ACVR1*, *ACVR1B*, *ACVR2A*, *ACVR2B* and *ERFE* genes (Table S2). The samples were amplified (7900HT Sequence Detection System; Applied Biosystems, Foster City, CA, USA) using standard cycling conditions. The primers were designed with the Primer Express 2.1 software (Applied Biosystems, Foster City, CA, USA). β-*Actin* and glyceraldehyde 3-phosphate dehydrogenase (*GAPDH*) were used as internal controls. Relative gene expression was calculated using the 2^−ΔCt^ method [25], with the fold change determined using the ratio between the gene expression and the internal control.

### 4.5. Protein Expression Analysis

#### 4.5.1. Cell Extracts

Proteins were extracted from the cells using RIPA lysis buffer in the presence of a protease inhibitor cocktail (Roche, Rotkreuz, Switzerland). Total protein extracts were analyzed by SDS–PAGE, transferred to polyvinylidene difluoride membranes (BioRad, Milan, Italy), and then incubated with the required combinations of the following antibodies: rabbit anti-GDF11 (1:500; ab124721; Abcam, Cambridge, UK); rabbit anti-SEC23B (1:500; SAB2102104; Sigma Aldrich, Milan, Italy); mouse anti-GATA1 (1:500; H00002623-M06; Abnova, Taipei, Taiwan); rabbit anti-pSMAD2 (1:500; 43108; Cell Signaling Technology, Danvers, MA, USA); rabbit anti-SMAD 2/3 (1:1000; 95678; Cell Signaling Technology); mouse anti-HSP70 (1:5000; SAB4200714; Sigma Aldrich, Milan, Italy); rabbit anti-SMAD4 (1:1000; ab215968; Abcam, Cambridge, UK); and rabbit anti-FAM132B (1:200; NBP2-57732; Novus Biologicals, Centennial, CO, USA). Rabbit anti-GAPDH (1:1000; 2118, Cell Signaling Technology, Danvers, MA, USA) and anti-TATA binding protein (TBP; 1:1000; ab51841; Abcam, Cambridge, UK) antibodies were used as the controls for equal loading of total and nuclear protein. Semi-quantitative analysis of protein expression was performed as previously described [26]. The bands were quantified using the Quantity One software (BioRad, Milan, Italy), to obtain integrated optical densities, which were then normalized to GAPDH or TBP.

#### 4.5.2. Secreted Proteins

Expression of secreted proteins was analyzed by loading plasma samples (from the healthy controls and patients) onto SDS–PAGE, followed by transfer to polyvinylidene difluoride membranes and incubation with the anti-GDF11 antibody (1:500; ab124721; Abcam, Cambridge, UK). The data were normalized through Ponceau red staining of the blots.

### 4.6. Subcellular Fractionation

Subcellular fractionation of the nuclear and cytosolic proteins was performed according to the Schreiber method [27]. Briefly, the harvested cells were washed twice with ice-cold phosphate-buffered saline and homogenized in ice-cold buffer (10 mM HEPES, pH 7.9, 1.5 mM MgCl2, 1 mM EDTA, 0.5 mM dithiothreitol, 10% [*v*/*v*] glycerol, 1 mM phenylmethylsulfonyl fluoride and protease inhibitor cocktail [Roche, Rotkreuz, Switzerland]). The suspensions were then repeatedly passed through the needle (26 gauge) of a syringe and then centrifuged at 800× *g* for 5 min at 4 °C to separate the cytosol from the nuclear pellet. The nuclear pellet was resuspended in the same lysis buffer in the presence of 3 M KCl, with this nuclear extract stored in ice for 1 h and then centrifuged at 16,000× *g* for 30 min at 4 °C.

### 4.7. Statistical Analysis

The statistical significances of the differences in protein and gene expression were assessed using Student’s *t*-tests or Mann–Whitney tests. Statistical significances of multiple comparisons were calculated using ANOVA, and post-hoc correction was performed using Tukey’s multiple comparison tests. A two-sided *p* ≤ 0.05 was considered statistically significant.

## Supplementary Materials

Supplementary materials can be found at https://www.mdpi.com/1422-0067/21/15/5577/s1.

## Abbreviations

CDA II	Congenital Dyserythropoietic Anemia type II
GDF11	Growth Differentiation Factor 11
CDAs	Congenital dyserythropoietic anemias
ERFE	Erythroferrone
TGF-β	Transforming Growth Factor beta
GDFs	Growth Differentiation Factors
BMPs	Bone Morphogenetic Proteins
ActR	Activin Receptors
HCs	Healthy Controls
CTR	Control
NT	Non-treated
Hbb	Hemoglobin subunit beta
