# Supplementary material for: RAP-011 Rescues the Disease Phenotype in a Cellular Model of Congenital Dyserythropoietic Anemia Type II by Inhibiting the SMAD2-3 Pathway
Source: Int J Mol Sci. 2020 Aug 4;21(15):5577. doi: 10.3390/ijms21155577 (PMC7432210; doi:10.3390/ijms21155577)
Supplement: Supplementary file 1 [file ijms-21-05577-s001.pdf]

Supplementary file

RAP-011 rescues the disease phenotype in a cellular model of congenital dyserythropoietic anemia type II by inhibiting the SMAD2-3 pathway

Gianluca De Rosa,[1,2] Immacolata Andolfo,[1,2,*] Roberta Marra,[1,2] Francesco Manna,[1,2] Barbara Eleni Rosato,[1,2] Achille Iolascon[1,2] and Roberta Russo[1,2,*]

[1]Department of Molecular Medicine and Medical Biotechnologies, University of Naples Federico II, Naples, Italy
[2]CEINGE Biotecnologie Avanzate, Napoli, Italy

Running title: RAP-011 treatment in CDA II cellular model

***Corresponding authors:**
Roberta Russo, PhD
Dipartimento di Medicina Molecolare e Biotecnologie Mediche
Università degli Studi di Napoli Federico II
CEINGE – Biotecnologie Avanzate
Via Gaetano Salvatore, 486
80145 Naples (Italy)
Email: roberta.russo@unina.it
Tel: +39-081-3737736

Immacolata Andolfo, PhD
Dipartimento di Medicina Molecolare e Biotecnologie Mediche
Università degli Studi di Napoli Federico II
CEINGE – Biotecnologie Avanzate
Via Gaetano Salvatore, 486
80145 Naples (Italy)
Email: andolfo@ceinge.unina.it
Tel: +39-081-3737736

Table of contents:

- Table S1
- Table S2
- Figure S1
- Figure S2
- Figure S3

Table S1. Clinical features of the patients with CDA II included in this study.

Patient ID	Country of origin	Age at diagnosis (years)	RBC count (10^6/µL)	Hb (g/dL)	Ht (%)	MCV (fL)	MCH (pg)	MCHC (g/dL)	RDW (%)	Platelet count (10^3/µL)	Absolute reticulocyte count (/µL)	Total bilirubin (mg/dL)	Unconjugated bilirubin (mg/dL)	Ferritin (ng/mL)	Transferrin saturation (%)	Reference Pubmed ID
NR33	Italy	43	3.34	10.5	31.3	93.9	31.6	33.5	-	286	90.0	4.9	3.8	437	95	21850656
NR54	Italy	10	3.23	9.5	26.1	83.4	29.0	35.2	-	-	42.3	3.4	2.8	206	58	20941788
NR61	Italy	7	3.89	10.2	30.6	79.0	26.2	33.2	21.1	294	58.4	1.9	1.3	119	52	25044164
NR63	Italy	35	3.41	10.1	31.0	91.1	29.7	32.6	21.9	706	35.0	0.8	0.6	1883	112	25044164
iNR43	USA	18	2.96	11.6	34.0	114.0	39.2	34.3	18.6	1065	32.6	4.8	4.3	216	99	25044164
iNR51	Turkey	8	3.36	9.5	27.5	81.8	28.3	34.6	20.7	474	26.9	1.8	1.5	25	-	25044164
iNR52	Turkey	10	2.40	6.7	20.3	84.7	27.9	32.9	24.3	415	19.2	-	-	100	-	25044164
iNR53	Turkey	7	2.09	6.1	18.3	87.0	29.4	33.7	13.7	232	150.5	2.5	1.9	1218	-	25044164
iNR54	Greece	4	2.85	8.9	27.0	94.7	31.3	33.1	20.1	281	83.5	1.7	1.2	64	61	25044164
iNR56	Turkey	13	3.57	11.7	33.5	94.0	31.5	33.5	14.0	266	164.2	4.6	3.8	-	-	27540014
iNR58	Turkey	15	3.59	11.0	30.8	93.2	33.3	35.1	15.2	256	82.6	3.1	2.9	46	-	27540014
iNR62	Greece	27	3.00	9.9	29.9	92.0	33.0	-	-	187	105.0	4.2	3.9	117	-	27540014

RBC, red blood cell; Hb, hemoglobin; Ht, hematocrit; RDW, RBC distribution width.

Table S2. List of primers used in the study

Primers used in qRT-PCR analysis		
**Gene**	**Forward Primer Sequence** (5’ → 3’)	**Reverse Primer Sequence** (5’ → 3’)
ABCB6	TCTGGCTGCATCCGAATAGAT	GGGCACAACTCCAATGTGAGA
ACVR1	ACTGTTGGAGTGTGTGTCGG	CAACATTCTCCCCTTGCC
ACVR1B	GAGGAAATGCGAAAGGTTGT	CCGCAGTGCCTCATAACT
ACVR2A	GCTGTGAGGGCAATATGTGT	GATTTGAAGTGGGCTGTGTG
ACVR2B	AGGCAACTTCTGCAACGAAC	TGGCTCGTACGTGACTTC
ALAS2	CAGTTCCTGTTTGGTATTGGACG	TGCCTTCTGCACAATCTTGCT
BAD	CGGAGGATGAGTGACGAGTT	GGAGCTTTGCCGCATCTG
BAX	TGCAGAGGATGATTGCCG	GTTGCCGTCAGAAAACATG
BCL-2	GCCCTGTGGATGACTGAGTA	GGCCGTACAGTTCCACAAAG
ERFE	CAGTGAGCTCTTCACCATCT	TCCAAGAACACGGAGGTC
GAPDH	CCACATCGCTCAGACACCAT	AGTTAAAAGCAGCCCTGGTGAC
HBB	GTGGATCCTGAGAACTTCAGGC	TCTTTGCCAAAGTGATGGGC
HBG	GGAGATGCCACAAAGCACCTG	AAACGGTCACCAGCACATTTCC
KLF1	TTGCGGCAAGAGCTACACC	ACGTGCAGGCGTATGGCT
SEC23A	CAGTATCAGCATTCAAGTGGG	AAAGATGCAGGAATGTTTTGGAT
SEC23B	GGTCCATGCGTATCTCTGAATG	CACTGACTCGTGCCACCAACA
Primers used in SEC23B silencing		
Sh-*SEC23B*-70 (#V3LHS_357970)	GCATTAAAGCAGCGTATC	-
Sh-*SEC23B*-74 (#V3LHS_357974)	TGCACAACACTTCATCTCC	-

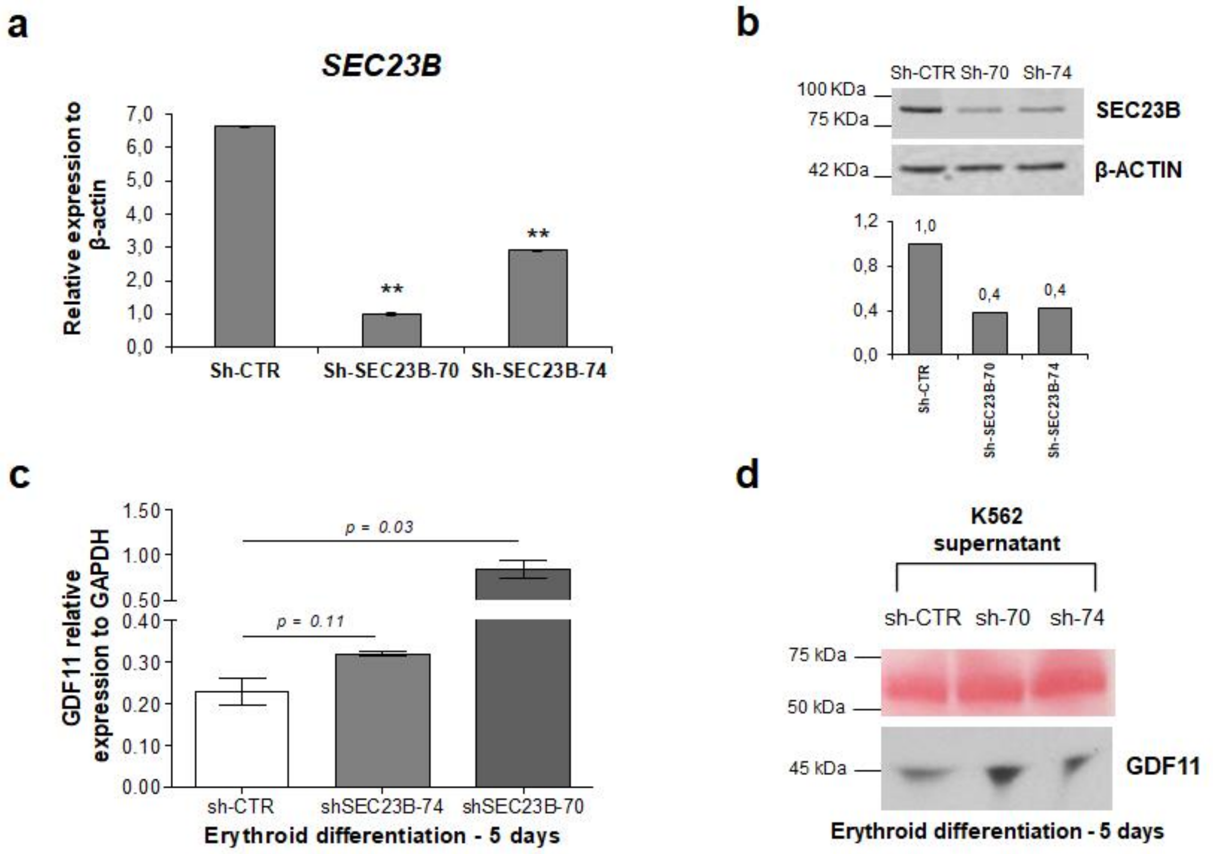

Figure S1. K562 sh-SEC23B-70 and -74 establishment, and *in-vitro* analysis of GDF11 expression.
(a) Relative *SEC23B* expression in K562 sh-CTR, K562 sh-SEC23B-70 (sh-70) and K562 sh-SEC23B-74 (sh-74) cells. Data are means ±standard deviation. *p < 0.05; **, p <0.01 (Student t-tests). **(b)** Representative western blot of SEC23B for K562 sh-CTR, K562 sh-SEC23B-70 (sh-70), and K562 sh-SEC23B-74 (sh-74) cells. **(c)** qRT-PCR for *GDF11* expression in K562 sh-CTR, K562 sh-SEC23B-70, and K562 sh-SEC23B-74 cells at 5 days of hemin treatment. Data are means ± standard deviation (Student t-tests). **(d)** Representative immunoblot of GDF11 in medium from K562 sh-CTR, K562 sh-SEC23B-70 (sh-70), and K562 sh-SEC23B-74 (sh-74) cells at 5 days of hemin treatment. Normalization of GDF11 protein was through Ponceau red staining of the blots.

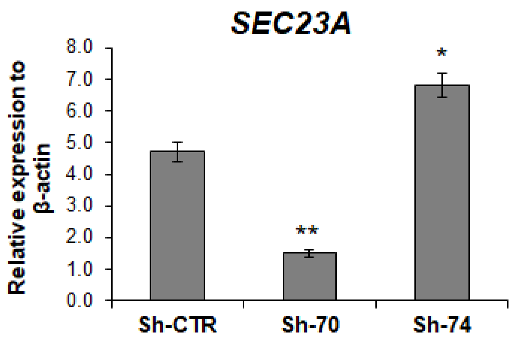

Figure S2. Analysis of SEC23A gene expression in K562 cells stably silenced for *SEC23B.*
Relative *SEC23A* expression in K562 sh-CTR, K562 sh-SEC23B-70 (sh-70) and K562 sh-SEC23B-74 (sh-74) cells. Data are means ±standard deviation. *p < 0.05; **, p <0.01 (Student t-tests).

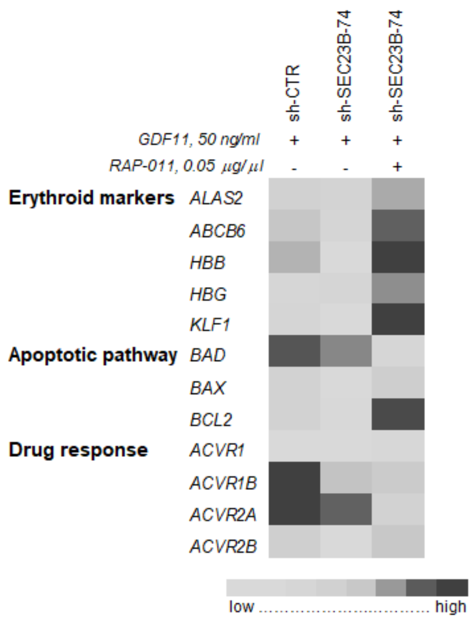

Figure S3. Gene expression profiling in RAP-011–treated cells.
Heat map for expression profiling for each of the cell clones (as indicated) treated with GDF11 or with GDF11+RAP-011. Fold-changes for sh-CTR and sh-SEC23B-74 cells treated with GDF11 were calculated relative to cells treated with vehicle. Fold-changes for sh-SEC23B-74 cells treated with GDF11+RAP-011 were calculated relative to sh-SEC23B-74 cells treated with GDF11. Gene expression: light gray, low; grey, medium; dark gray, high.
